# Quantifying Host Potentials: Indexing Postharvest Fresh Fruits for Spotted Wing Drosophila, *Drosophila suzukii*

**DOI:** 10.1371/journal.pone.0061227

**Published:** 2013-04-12

**Authors:** David E. Bellamy, Mark S. Sisterson, Spencer S. Walse

**Affiliations:** San Joaquin Valley Agricultural Sciences Center, United States Department of Agriculture–Agricultural Research Service, Parlier, California, United States of America; Michigan State University, United States of America

## Abstract

Novel methodology is presented for indexing the relative potential of hosts to function as resources. A Host Potential Index (HPI) was developed as a practical framework to express relative host potential based on combining results from one or more independent studies, such as those examining host selection, utilization, and physiological development of the organism resourcing the host. Several aspects of the HPI are addressed including: 1) model derivation; 2) influence of experimental design on establishing host rankings for a study type (no choice, two-choice, and multiple-choice); and, 3) variable selection and weighting associated with combining multiple studies. To demonstrate application of the HPI, results from the interactions of spotted wing drosophila (SWD), *Drosophila suzukii* Matsumura (Diptera: Drosophilidae), with seven “reported” hosts (blackberries, blueberries, sweet cherries, table grapes, peaches, raspberries, and strawberries) in a postharvest scenario were analyzed. Four aspects of SWD-host interaction were examined: attraction to host volatiles; population-level oviposition performance; individual-level oviposition performance; and key developmental factors. Application of HPI methodology indicated that raspberries (*^mean^HPI_varied_* = 301.9±8.39; rank 1 of 7) have the greatest potential to serve as a postharvest host for SWD relative to the other fruit hosts, with grapes (*^mean^HPI_varied_* = 232.4±3.21; rank 7 of 7) having the least potential.

## Introduction

There is interest in many areas of biology to understand and define relations between a host and the organism it harbors. Host preference and host suitability, as generally termed, are both critically related to the potential of a host to serve as a resource [1]–[3]. When evaluating a suite of hosts, indices offer a mathematical framework to quantitatively integrate findings from multiple types of host suitability and host preference studies into an overall expression of host potential. Yet, very little research has been dedicated towards this end.

Host preference studies examine behaviors associated with host selection (e.g., host location, distribution, and abundance) [4]–[6] at individual and population levels [7]–[9]. While various indices have been developed for host preference studies, most are species or system specific (“Feeding Index” [10]; “Human Blood Index” [11]; “Preference Index” [12]). Some preference indices have broader applications such as those developed to enumerate the relative behavioral response of individuals during host selection [7], evaluate preference as proxied by mark-release-recapture ratios [13], or quantify preferential utilization based on host spatiotemporal considerations [14].

Host suitability studies evaluate factors affecting the physiological development/performance of the harbored organism and other features of host quality [15]–[18]. Host suitability studies using indices are also rare, but have been applied to some biological systems. For example, larval performance can be evaluated using the Egg Production Index (EPI) [19], which estimates egg production based on pupal mass. Perhaps the most comprehensive application is the “Host Suitability Index” [20] which uses pupal weight, leaf consumption, development time, and survival rate to calculate host suitability for herbivorous insects.

There are several rating systems capable of producing ranking indices that have not been applied to host preference or host suitability. For example, several vertebrate behavior studies looking at paired outcomes (e.g., male **A** vs. male **B** dominance) [21]–[23] use the Elo-rating method [24], which was originally developed as an unbiased tool for ranking chess players. The Elo model has several advantageous features, including flexibility in the number of contestants which may be updated from event to event or through time [22]. Additionally, there is the Data Envelopment Analysis (DEA) [25] method, which is used for determining preference and ranking in multiple-choice outcomes such as political voting analyses.

The purpose of this research is to develop a practical index to quantify relative host potentials based on analyses of host preference and host suitability, regardless of the experimental design employed (e.g. no-choice, two-choice, or multiple choices). Accordingly, the Host Potential Index (HPI) was developed and applied to a scenario where understanding the relation between hosts and the harbored organism are of contemporary economic consequence in North America

The spotted wing drosophila (SWD), *Drosophila suzukii* (Matsumura), (Diptera: Drosophilidae) is a polyphagous insect associated with a variety of economically important hosts, including berry crops and stone fruits [26], [27]. SWD are good flyers capable of traveling over relatively large horizontal and vertical distances [28]. They possess a serrated ovipositor which allows them to exploit ripe fruit not normally associated with other drosophilids, which tend to prefer rotting or damaged hosts [29]. Due to the number of commercially-produced crops that might serve as possible hosts, the development of a host potential index for SWD assists scientists, agricultural practitioners, and regulators working within this system in prioritizing research and control efforts for each crop.

## Methods and Materials for Host Potential Index

The HPI model is used to calculate relative host potentials based on results from host suitability and/or preference studies that use no choice, two-choice, or multiple-choice experimental designs. A scoring system is applied and host rankings are subsequently established for each host in each study. The rankings of each host are then referenced against an index of tabulated HPI values. When hosts across studies are equivalent, the study-specific HPI values for each host can be pooled and prioritized using an additive model to yield on overall relative potential, *^tot^HPI_β_*, for each host.

### Data Collection, Scoring System, and Host Ranking for each Study

Data can be collected from studies that include no-choice [30], [31], two-choice [32], [33], and multiple-choice designs [34], [35]. Scoring and ranking procedures for respective study designs are described below.

#### No-choice Studies (Rank Order)

For no-choice studies, hosts are ranked by the overall means of the measured response with the most positive host response ranked first. If data examined by multi-sample analyses (e.g. ANOVA) followed by multiple comparison tests (e.g., Tukey-Kramer HSD) are used and result in means that are not statistically different, the ranks are averaged together. For example, a study measuring the mean adult mass of newly emerged females developing on three different hosts may find females are largest when developing on host **B**, followed by hosts **A** and **C** equivalently. Host **B** would have a rank of 1, **A** = 2.5, and **C** = 2.5.

#### Two-choice Studies (C-Score)

Two-choice studies involve three or more potential hosts each matched pairwise against one another (e.g., **A**∶**B**, **B**∶**C**, **A**∶**C**) where it is not uncommon for **A** to be preferred over **B**, and **B** to be preferred over **C**, but where **C** is preferred over **A**. To determine which host is most likely preferred in these situations (which cannot be distinguished by ANOVA), a method was developed that incorporates strengths of preference and results in a *C-score* for each host (refer to Supporting Information S1 for detailed example). The resulting *C-scores* may then be used to differentiate host preference in pairwise events. Initially, all unexamined hosts begin with a default C-score, *C* = 900. C-scores change after differences in preference probabilities are evaluated, as described below, for each pairing of potential hosts (A and B, B and C, etc.).

The *actual* preference probability for Host A, *A_a_*, is calculated from
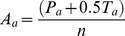
(1)Where *P_a_* is the number of times host **A** was preferred over host **B** in *n* pairwise trials, and *T_a_* is the number of occasions where no preference was observed (equal responses). Note that all variables pertaining to Host A are denoted by a subscripted “*a*,” and Host B by a subscripted “*b*.”

The *expected* preference probability for Host A, *E_a_*, is calculated from 
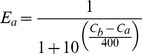
(2)Where *C_b_* is the current C-score of host **B**, and *C_a_* is the current C-score of host **A**. Thus, if the C-scores are equivalent, the expected probability for host **A** preference is 0.50. If *C_b_*−*C_a_*>400 the expected preference probability (*E_a_*) for host **A** is limited to 0.08, or an 8% chance of host **A** being favored; likewise, if *C_b_−C_a_*<−400, *E_a_* is capped at 0.92 to reflect the lack of absolute certainty due to chaos inherent in biological systems [36].

Once the actual and expected preference probabilities are calculated, the C-score for each potential host can be derived using a modified Elo-rating formula [24] that takes the number of pairwise events into consideration. 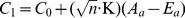
(3)Where *C_0_* is the C-score of host **A** prior to the pairwise combination, *n* is the number of pairwise events evaluated between host **A** and **B**, K is a constant determined by the K-factor table (Table 1), *A_a_* is the actual preference probability of host **A** in the pairwise combination **A∶B** (from Eq. 1), and *E_a_* is the expected preference probability of host **A** when paired with **B** (from Eq. 2). The K-factor term represents the number of points available to host **A** (adjusted by the number of trials) for increasing or decreasing its C-score. After calculation, *C_1_* becomes the new C-score for host **A** and should be used in the subsequent pairwise calculation. Note that the C-score for host **B** must be calculated separately in the same manner. To prevent scores from attaining a negative value, a floor of *C* = 100 was established. No maximum value was set for C-scores.

**Table 1:** C-scores and K-factors.

Min C-score	K-factor
100	5
162	7
222	10
278	12
329	14
377	16
420	18
460	19
496	21
530	23
560	24
588	25
614	27
638	28
660	29
680	30
699	31
718	32
735	33
752	34
786	35
840	36
900	36
986	35
1092	34
1180	33
1230	32
1284	31
1343	30
1407	29
1475	28
1549	27
1629	25
1715	24
1806	23
1905	21
2009	19
2121	18
2240	16
2367	14
2501	12
2643	10
2794	7
2953	5

Minimum C-scores and the corresponding K-factor for two-choice studies (Eq. 3). All untested hosts initially begin with a C-score = 900 and K-factor = 36 (Bold print) prior to being evaluated against another host.

To limit potential variation in scores due to pairing order, the host with the highest *overall* actual preference probability, *A*, (designated as Host 1) was initially paired with the host that had the lowest *overall* actual preference probability, and Eq. 3 was used to calculate the scores for each host. Subsequently, Host 1 was matched with the host possessing the second lowest actual preference probability (*A*) and *C*-scores were calculated for both hosts, until Host 1 was paired with every host in the study (and C-scores calculated). Computation proceeded as above, without Host 1, for the host with the second highest actual preference probability (Host 2). Evaluation continued until the two least preferred hosts were paired. C-scores for each host were then used to assign rank.

#### Multiple-choice Studies (M-Score)

When insects choose between several hosts simultaneously, dilution effects, where host **A** might influence selection between hosts **B** and **C**, must be taken into consideration. Data from multiple-choice studies was evaluated with a modified DEA formula originally used for voting analysis [25] and resulted in an M-score for each host examined (refer to Supporting Information S2 for detailed example). Results (considered here as “votes of preference”) from each replicate of the study were initially ranked and tabulated. For illustrative purposes, consider the total number of eggs oviposited on three different hosts (A, B, & C) exposed concurrently to individual females in four replicated trials: **A**, 24, 12, 20, and 18; **B**, 21, 21, 18, 17; and **C**, 23, 20, 19, and 16. The tabulated votes of preference would appear as in Table 2, with the most preferred host having the highest egg count in each trial. Because first place “votes” outrank 2^nd^ place, which in turn both outrank 3^rd^ place, etc., a weighting factor is assigned to each place, as calculated from

**Table 2:** Votes of Preference.

	1^st^ place ranks	2^nd^ place ranks	3^rd^ place ranks
Host A	2	1	1
Host B	1	0	3
Host C	0	3	1

Total number of eggs deposited in host A = 24, 12, 20, and 18; host **B**, 21, 21, 18, 17; and host **C**, 23, 20, 19, and 16.

“Votes of preference” for a hypothetical multiple-choice study involving four host selection behavior trials of individual females when concurrently exposed to three hosts.



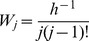
(4.)Where *W_j_* is the *j* ^th^ place weighting factor, *h* is the number of hosts in the study, and *j* is the place (1^st^, 2^nd^, etc.). The weighting factor conforms to the rule that *W_1_*≥2*W_2_*≥3*W_3_*≥*jW_j_*. For the example above, *W_1_* = 0.333, *W_2_* = 0.166, and *W_3_* = 0.055. The M-score is then calculated using the modified DEA formula



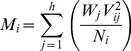
(5)Where *M_i_* is the M-score for host *i*, *V_ij_* is the number of *j* ^th^ place “votes of preference” for host *i*, and *N_i_* is the total number of “votes of preference” given to host *i*. Once M-scores are calculated, hosts are arranged by rank. If similar M-scores exist, ranks of those hosts would be averaged together. In the example (Table 2), *M_A_* = 0.388, *M_B_* = 0.207, and *M_C_* = 0.387. Host **A** is the preferred host in this study, (additional replications would likely be appropriate to resolve the slight difference between hosts **A** and **C)**. Host **B** would be ranked 3^rd^.

It is critical to note the utility of Eq. 5 for distinguishing preference in multiple-choice studies. Given the numerical example above, if the mean eggs per trial were used to establish rank (Host **A** = 18.5 eggs/trial; Host **B** = 19.3 eggs/trial; Host **C** = 19.5 eggs/trial), the results would be markedly different. Furthermore, an ANOVA identifies no differences in rank.

### Tabulating HPI values

Once ranks are calculated for each study, the corresponding Host Potential Index value can be determined by referencing the HPI table (Table 3), which lists columnar values corresponding to the number of hosts in the study and row values of rank. HPI scores are denoted with a precedent superscript describing the HPI value (e.g., total, test 1, flight, etc.) along with subscripts for the weighting system, *β* (*vide infra*), *^test^HPI_β_*. The reported value is then followed by the rank of that host followed by the number of hosts examined (e.g., using 4 equally weighted studies, a rank of 3 out of 10 hosts would result in an *^tot^HPI_equal_* = 316; rank 3 of 10). Indexed values were derived through an iterative process and bounded such that no value reflected a “zero” potential while still providing a spread of values positively correlated to both rankings and the number of hosts evaluated (e.g., 2^nd^ of 14 hosts results in a stronger host potential than being 2^nd^ of 5 hosts) (refer to Supporting Information S3 for additional information). If ties exist in rank order, then ranks of those hosts are averaged together. For example, if 6 hosts were examined and their ranks were 1, 2, 3.5, 3.5, 5 and 6, the HPI for the shared ranks of 3.5 would be mid-way between 271 and 257 (or 264) for both hosts.

**Table 3:** Host Potential Index.

HOST POTENTIAL INDEX																			
Rank of host	number of hosts																		
*R_n_*	2	3	4	5	6	7	8	9	10	11	12	13	14	15	16	17	18	19	20
**1**	263	276	289	303	316	329	342	355	368	382	395	408	421	434	447	461	474	487	500
**2**	239	251	263	274	286	298	310	322	334	345	357	369	381	393	405	417	428	440	452
**3**		238	249	260	271	283	294	305	316	327	339	350	361	372	383	394	406	417	428
**4**			236	246	257	267	278	288	299	309	320	330	341	351	362	372	383	394	404
**5**				232	242	252	262	271	281	291	301	311	321	331	341	350	360	370	380
**6**					227	236	245	255	264	273	282	292	301	310	319	328	338	347	356
**7**						221	229	238	246	255	264	272	281	289	298	306	315	323	332
**8**							213	221	229	237	245	253	261	269	276	284	292	300	308
**9**								204	212	219	226	233	241	248	255	262	270	277	284
**10**									194	201	207	214	221	227	234	240	247	253	260
**11**										183	189	195	200	206	212	218	224	230	236
**12**											170	175	180	186	191	196	201	207	212
**13**												156	160	165	170	174	179	183	188
**14**													140	144	148	152	156	160	164
**15**														124	127	130	133	137	140
**16**															105	108	111	113	116
**17**																86	88	90	92
**18**																	65	67	68
**19**																		43	44
**20**																			20

The Host Potential Index indicates the strength of the organism-host relationship. Values range from 20 (low host potential) to 500 (high host potential) for 20 theoretical hosts and could be extrapolated to include an unlimited number of simultaneously examined hosts.

### Pooling and Prioritizing HPI values

If more than one study is considered when evaluating relative host potential, an overall HPI can be derived as a weighted total, (*^tot^HPI_β_*), in the form 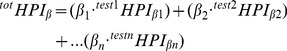
(6)Where *β_1_* is the weighting coefficient for study 1, *β_2_* the coefficient for the study 2, and *β_n_* is the coefficient for the nth study, and Σ*β_x_* = 1.00. The model's multiplicative coefficient terms allow flexibility in prioritizing the contribution of a single study toward the overall evaluation of relative host potential, while the additive function precludes a “near-zero potential” in one study from obscuring results in other studies that support greater relative host potentials.

## Methods and Materials for Case Study: Swd Host Potential

The following application of the HPI involves spotted wing drosophila, where *^lp^HPI_β1_* references the Larval Performance Study (no-choice design), *^flt^HPI_β2_* the Flight Bioassay (two-choice design), *^pop^HPI_β3_* the Population Level Oviposition Study (multiple-choice design), and *^ind^HPI_β4_* the Individual Level Oviposition Study (multiple-choice design).

### Spotted Wing Drosophila Colony and Potential Host Fruits for All Studies

SWD pupae were originally obtained from the laboratories of Dr. Arytom Kopp (UC Davis) and Dr. Jana Lee (USDA-ARS) and colonies were established at the USDA-ARS-SJVASC (Parlier, California) as described in Walse et al. [37]. Blueberries, blackberries, sweet cherries, table grapes, raspberries, strawberries, and yellow peaches (Size 72) were of high-quality and obtained postharvest from commercial sources in California USA and stored at 1.1 °C±1.4 °C (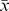
±*s*).

### No-Choice Study (Rank Order): Larval Performance

#### Diet

Media consisted of a fruit and agar mixture an were prepared as follows. Blueberries, blackberries, cherries, grapes, peaches, raspberries, or strawberries, were quickly dipped into a 35% ethanol bath, removed, and washed thoroughly in DI water. Each fruit type was respectively pureed in a laboratory blender and 400 mL of fruit pulp and juice, as well as 2 mL of formalin fungistat, were added to 400-mL of standard cornmeal-(dextrose or sucrose)-agar-yeast medium [37] before set. The resulting ∼1∶1 fruit-agar media was poured into 100×15-mm polystyrene Petri dishes to a depth of 6.0±0.6 mm (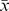
±*s*) and allowed to solidify.

Population density is known to have an effect on development in some insects [38], so multiple linear regression analyses were conducted to probe potential relationships in this experimental design. In a series of trials independent of this HPI study, SWD developing on whole blackberries (*F_1,15_* = 5.610, *P* = 0.032), grapes (*F_1,11_* = 6.948, *P* = 0.023), and raspberries (*F_1,15_* = 10.625, *P* = 0.005) did exhibit density dependent effects on development time, but these were not observed when SWD developed on the fruit-agar media (blackberry, *F_1,3_* = 1.581, *P* = 0.298; grape, *F_1,10_* = 0.009, *P* = 0.926; and raspberry, *F_1,4_* = 0.057, *P* = 0.823). Additionally, the fruit-agar media provided a surface-area normalized presentation for each fruit type thereby reducing the potential for confounding variables associated with fruit morphology and oviposition behavior.

#### Infestation

Four nylon-mesh enclosures (Bug Dorm-2®, BioQuip Products, Rancho Dominguez, CA, USA), containing ∼2000 adult SWD each, were transferred from the rearing chamber to a 14.0-m^3^ infestation chamber (24–27 °C, 80% RH, 16∶8 [L∶D] h). Each enclosure housed replicate Petri dishes (two) of blueberry, blackberry, cherry, grape, peach, raspberry, and strawberry agar media. All plates were introduced and lids were removed to initiate exposure to SWD. After a 24-h ovipositional period, Petri dishes were re-covered with lids, removed from the enclosure, labeled, and transferred to a 12.3-m^3^ incubation unit (24–27 °C, 80% RH, 16∶8 [L∶D] h). Petri dishes were examined daily for adult SWD emergence. All emerging adults (<1-d old) were aspirated off the media, counted, and recorded [37]. Subsamples of randomly-selected females were placed into source-labeled vials containing Insect Ringer's solution (per liter of H_2_O: 10.93-g NaCl, 1.57-g KCl, 0.83-g CaCl_2_*2H_2_O, 0.83-g MgCl_2_*6H_2_O) for gravimetric determination of mass. (*vide infra*).

#### Performance analysis and developmental score

Larval performance was quantified, and rank for each host subsequently established, by combining three developmental diagnostics into a developmental score, *D*, in the form
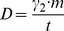
(7)where *γ_2_* was the kurtosis of the distribution of daily adult emergence, *m* was the mean mass of newly-eclosed adult females, and *t* was the mean time for adult emergence. The magnitude of *D* in Eq. 7 varies directly with host quality [3].

A histogram of adult emergence was generated for each fruit-agar dish and the mean time for adult emergence (*t*) and associated kurtoses, *γ*_2_, were calculated from the resulting distributions. A random sub-sample of 10 females was taken from each stored vial of Insect Ringer's solution (containing>50 SWD females) and weighed in bulk on an Ohaus Explorer microbalance (model #E04130) and average female mass (*m*) was determined for each variety of fruit-agar. The 10 females were returned and another sub-sample of 10 females was randomly chosen from the same vial and weighed. This process was repeated five times for each type of fruit. Mean D-scores (Eq. 7) for each fruit-agar variety were calculated and ranked.

### Two-Choice Study (C-Score): Flight Bioassay

#### Flight Chambers

Two Plexiglas flight chambers (0.61×0.61×2.13 m) (Analytical Research Systems, Gainesville, FL, Model #OLFM-WT-24×24×84) were used concurrently. Both flight chambers were oriented north to south in a shaded greenhouse at the USDA-ARS-SJVASC in Parlier, CA maintained at 20–30 °C and 60±5% RH (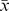
±*s*). Each chamber was fitted with a Dayton blower (Grainger, Model #4C119) powered by a 1118.55-W motor that drew air through the flight chamber and vented the exhaust external to the greenhouse. The air intake end of the flight chambers were furnished with three charcoal filters and one Filtrete® air filter (61×61×2.5 cm) (1900 MPR or 13 MERV rating) which was duplicated on the exhaust end of the chambers. Release platforms consisting of ring stands with adjustable angle flat-clamps were placed centrally 0.4 m from the rear and above the floor of each chamber. Each of two recapture traps were comprised of a 30.5-cm length of L-shaped 5.0-mm (I.D.) Pyrex® glass tubing connected to a 111-mL polystyrene sampling cup by a threaded polyethylene connector placed through the bottom of the sampling cup, thereby orienting the length of the cup horizontally and parallel to the wind direction. The traps on each chamber were located 22.8 cm from the edge of the lateral chamber walls and 25 cm downwind from the air intake. A double-sided yellow sticky card (Alpha Scents, Portland, OR) cut to 38×70 mm was inserted into each trap to immobilized insects. Finally, each trap was sealed with a lid having a 9.5-mm hole drilled centrally to allow passage of the volatile-containing airstreams into the flight chamber and a threshold for test insects.

#### Volatile Collection and Delivery System

A modified volatile collection [39] and delivery system was attached to both flight chambers. Airflow was supplied by a compressor at 410 kPa and was pushed through two Varian Chrompack Gas-Clean moisture filters (Model #CP17971), an activated carbon filter, a humidifying bubbler, and a 6-channel air delivery system (ARS, Gainesville, FL, Model #VCS-ADS-6AFM6C) in series. Each of two channels allowed a 1.5-L/min airflow, which was directed through 6.35-mm Teflon® flex-tubing to the inlet port of separate volatile collection tubes (ARS, Gainesville, FL, Model #RV-A3) that each contained 100±2.0 mg (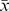
±*s*) of fruit. Airflow exited each collection tubes through two exit ports (exit ports 3 & 4 of each tube were plugged) and was directed through Teflon® flex-tubing to a trap on each flight chamber. In this manner, each flight chamber simultaneously received equal quantities of fruit volatiles from each collection tube for pairwise comparisons.

#### Flight Bioassay

To evaluate attraction to fruit volatiles, 80 mated female SWD (4 to 7-d old) were aspirated from the rearing colony and evenly separated into two 55.5-mL polystyrene vials, which were then capped and clamped to the release platforms 1 h prior to connecting the air delivery system to the flight chamber. Wind speed in the flight chambers was adjusted to 0.4±0.31 m/s (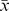
±*s*) to optimize flight conditions for drosophilids [40]. The vials were clamped to the platforms oriented upwind at 45° from perpendicular to the floor and then the caps were removed allowing the SWD to move freely within the flight chambers for 18 h (1500 to 0900). Subsequently, sticky cards were collected from traps, recaptured SWD counted and recorded, and flight chambers cleaned with a methanolic solution and allowed to air out before the next trial.

#### Flight Bioassay Analysis

The total number of recaptured female SWD for each fruit in the flight bioassay trials were used to calculate actual preference probabilities, *A* (Eq. 1). For this study, which involved seven hosts, a matrix containing *n*(*n*−1) cells was created (Table 4) and lists the actual preference probabilities from Eq. 1 of all pairwise combinations examined for each host. The results were used in Eqs. 2 and 3 to generate C-scores.

**Table 4:** Flight Bioassay Actual Preference Probabilities.

Fruit	Blackberry	Blueberry	Cherry	Grape	Peach	Raspberry	Strawberry
Blackberry		0.000	0.000	0.000	0.125	1.000	0.500
Blueberry	1.000		0.375	0.167	0.750	1.000	1.000
Cherry	1.000	0.625		0.375	1.000	0.750	0.250
Grape	1.000	0.833	0.625		0.500	1.000	1.000
Peach	0.875	0.250	0.000	0.500		1.000	1.000
Raspberry	0.000	0.000	0.250	0.000	0.000		.0250
Strawberry	0.500	0.000	0.750	0.000	0.000	0.750	
**Overall PCT**	**0.729**	**0.327**	**0.333**	**0.173**	**0.396**	**0.917**	**0.667**

Actual preference probabilities of paired flight bioassays for mated spotted wing drosophila females responding to host **A** volatiles (column) when simultaneously exposed to host **B** volatiles (row).

### Multiple-Choice Study 1: Population Level Oviposition

#### Infestation

Four nylon-mesh replicate enclosures, containing ∼2000 adult SWD each, were transferred to an infestation chamber maintained with rearing conditions as described above. Stainless-steel trays (30×30×2 cm) filled with monolayers of mixed fruit types (blackberry, blueberry, cherry, grape, peach, raspberry, and strawberry) were introduced into each enclosure. The stainless-steel trays containing infested fruit were removed from enclosures after a 72-hr ovipositional period. Infested berries were transferred to stainless-steel mesh ball cages (5.1-cm diameter) (2 blackberries/ball, 3 blueberries/ball, 2 cherries/ball, 3 grapes/ball, 3 raspberries/ball, and 0.75 strawberries/ball), while peaches were transferred to 473-mL glass jars with fine-screen lids. Mesh ball cages containing berries were grouped by replicate and berry type, placed inside pull-string cloth bags (25 per bag, per replicate), and transferred to an incubation unit (*vide supra*) along with the 4 replicates of 25 glass jars, each containing a peach sample. Glass jars (peach) and mesh ball cages were both removed from the incubation chamber daily and inspected for adults and fruit quality. If fruit showed signs of desiccation, an additional, uninfested berry or peach slice was added to provide moisture and/or resource. Live adults were removed, counted, and recorded.

#### Population Level Oviposition Analysis

For each replicate (*n* = 4) of 25 samples (steel-mesh balls or glass jars), average emergence from each fruit type was normalized relative to the surface area of the assayed unit (steel-mesh ball). Within each replicate, fruit types were ranked and designated/tallied with 1^st^, 2^nd^, 3^rd^, etc. place “votes of preference.” A steel-mesh ball was estimated to have 12% the surface area of a peach. Results were input into Eq. 5 to generate an M-score for each fruit host.

### Multiple-Choice Study 2: Individual Level Oviposition

#### Infestation

To investigate potential for oviposition to be impacted by a myriad of possibilities, such as leking or prior oviposition events [7], individual gravid females were exposed to all fruit types simultaneously. For each trial (*n* = 4), 25 mated SWD females (4–7 d) from the colony were placed into individually respective 3.79-L glass cages with mesh lids containing a single blackberry, blueberry, cherry, grape, peach, raspberry, and strawberry and housed in a 12.3-m^3^ incubation unit (24–27 °C, 80% RH, 16∶8 [L∶D] h). Females were removed after a 72-h ovipositional period and individual fruits were collected, labeled, and placed in mesh ball cages or jars as described above. Incubation and temporal analysis of adult emergence was as described in population level oviposition studies.

#### Individual Level Oviposition Analysis

Numbers of adults emerging from fruits were used to generate a table of tallied votes of preference (*vide supra*) and M-scores were derived using Eq. 5. If no adults emerged from any of the hosts exposed to a single female then that replicate was omitted from calculations since no fruit preference could be assigned for comparisons.

### Statistics and Simulations

For the no-choice study involving larval performance (D-score) an ANOVA, or if appropriate, a Welch's ANOVA, was used to rank each host based on the mean responses for each host. If the ANOVA was significant a Tukey-Kramer HSD with α = 0.05 was used to establish rank.

An algorithm was written in C++ (Microsoft Visual C++, 2010) to evaluate pairing order effects on C-scores using equation 3 and dummy data sets for a specified number of host pairings with fixed differences in host preference. The number of hosts in the dummy data set was varied from 2 to 10 and the number of paired comparisons (i.e., assays) was varied from 5 to 50. For each combination of hosts and pairwise assays, C-scores for each host were calculated for 100 random iterations. Quantifying the variation in C-scores based on paring order, number of hosts, and number of pairwise assays was used to estimate these effects on the generation of final scores. Due to the multiplicative nature of the *n* term in Eq. 3, variation in C-scores increased directly with number of pairwise assays. A scenario involving 50 pairwise events was used for all calculations and statistical analyses reported. Computer-simulated C-scores and the resulting variation in rank due to pairwise sequencing were examined using both multiple and simple linear regression analyses [41].

The effect of varying weighting coefficients on possible overall HPI values was examined using a four term simplex-lattice design with 231 distinct weighting scenarios [42] for each host with constraints limiting the terms (*β*_1_ to *β*_4_) to values between 0.000 and 1.000, while summing to 1.000. HPI values for each host and each study were placed into Eq. 6 for each simulated weighting scenario and corresponding overall *^tot^HPI_varied_* values were calculated for each host. An ANOVA, or if appropriate, a Welch's ANOVA examine possible shifts in overall relative host potential due to the weighting of coefficients. If the ANOVA was significant (*P*<0.05) a Tukey-Kramer HSD with α = 0.05 was used.

## Results and Discussion

### No-Choice Study (Rank Order): Larval Performance

Adult emergence varied between hosts (ANOVA: *F_(6, 77)_* = 2.407; *P* = 0.035) with a maximum trial mean of 189.2±23.6 (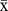
±*s*) for blackberry-agar medium and a minimum of 70.8±26.99 (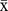
±*s*) for grape-agar medium. The overall mean time to peak adult emergence for all hosts following oviposition was 10.5±1.38 d (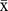
±*s*), which is 0.9 d longer than the mean oviposition to emergence time reported by Kanzawa [29] of SWD developing on cherries at 25 °C. Kanzawa's reported time of development on cherries, however, closely matched the peak time of emergence on the cherry-agar medium (9.7±0.42 d). The time to peak emergence varied between host-agar media (Welch's ANOVA: *F_(6, 25.94)_* = 5.303, *P* = 0.001). SWD larvae developed quickest on the cherry-agar medium and slowest on the grape-agar medium (12.1±1.93 d) (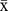
±*s*) (Table 5).

**Table 5:** Host Potential Summary for Spotted Wing Drosophila.

	Blackberry	Blueberry	Cherry	Grape	Peach	Raspberry	Strawberry
**Larval Performance**							
Kurtosis, *γ_2_*	5.13^B^	2.32^B^	6.54^A,B^	3.80^B^	10.80^A^	5.21^B^	4.66^B^
Adult fem mass (mg)	1.50^B^	1.48^B^	1.80^A^	1.44^B^	1.51^B^	1.83^A^	1.45^B^
Mean develop time (d)	10.2^B^	10.7^A,B^	9.7^B^	12.1^A^	10.3^A,B^	10.1^B^	10.9^A,B^
*D-score*	0.75	0.32	1.22	0.41	1.56	0.96	0.63
Rank	4	7	2	6	1	3	5
***^lp^HPI_β1_***	**267**	**221**	**298**	**236**	**329**	**283**	**252**
**Flight Bioassay**							
*C-score*	980.25	835.87	835.51	771.38	881.00	1038.13	957.86
Rank	2	5	6	7	4	1	3
***^flt^HPI_β2_***	**298**	**252**	**236**	**221**	**267**	**329**	**283**
**Population Oviposition**							
Mean SWD/Fruit	6.56^B^	1.90^C,D^	7.01^B^	0.43^D^	0.04^D^	5.39^B,C^	26.45^A^
% total emerging	18.6^B^	8.1^C^	19.9^B^	2.4^C^	0.0^D^	22.9^A,B^	28.1^A^
*M-score*	0.15	0.03	0.18	0.01	0.00	0.66	1.09
Rank	4	5	3	6	7	2	1
***^pop^HPI_β3_***	**267**	**252**	**283**	**236**	**221**	**298**	**329**
**Individual Oviposition**							
Freq. of Oviposition	0.58	0.57	0.23	0.26	0.00	0.60	0.72
Mean SWD/Fruit	1.32^B,C^	1.74^B,C^	0.89^C^	0.57^C^	0.00^C^	3.54^A,B^	5.11^A^
Standardized SWD/Fruit	2.63^B^	5.23^A,B^	2.66^B^	1.71^B^	0.00^B^	10.63^A^	5.11^A,B^
*M-score*	0.51	1.07	0.22	0.12	0.00	1.56	2.10
Rank	4	3	5	6	7	2	1
***^ind^HPI_β4_***	**267**	**283**	**252**	**236**	**221**	**298**	**329**
**Overall HPI**							
***^tot^HPI_equal_***	**274.9**	**251.7**	**267.2**	**232.4**	**259.4**	**301.9**	**298.1**
**Host Potential Ranking**	**3**	**6**	**4**	**7**	**5**	**1**	**2**

Row values not connected by the same letter are significantly different (Tukey-Kramer HSD, α = 0.05)

Summary of results for all four studies examining host potential for spotted wing drosophila.

The peakedness of adult emergence distributions (i.e., kurtosis) varied amongst types of fruit-agar media (Welch's ANOVA: *F_(6, 27.287)_* = 28.690, *P*<0.001). Peach- and cherry-agar diets had the highest kurtoses with the bulk of adults emerging over a span of 48-h (Table 5). However, no difference in kurtoses were observed across berry types (Tukey-Kramer HSD, *α* = 0.05), which displayed distributions that were generally long-tailed and spanned several days.

In *Drosophila* spp., several laboratory studies have shown a phenotypic correlation between adult body size and female fitness [43], [44], [45], [46], so female body size was used here as an indicator of diet quality. Newly emerged females from both the raspberry- and cherry -agar media were significantly larger than those emerging from other media (Welch's ANOVA: *F_(6, 12.24)_* = 17.221, *P*<0.001; Tukey-Kramer HSD, α = 0.05)(Table 5). The heaviest females developed on raspberry-agar medium (1.83±0.234 mg) (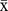
±*s*) and the lightest emerged from grape-agar (1.44±0.037 mg) (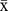
±*s*). When all three indicators of larval performance were considered in Eq. 7, peach-agar medium had the highest score (*D* = 1.56±0.22 mg/d) (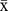
±*s*), whereas the grape-agar medium had the lowest score (*D* = 0.41±0.15 mg/d) (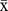
±*s*) (Welch's ANOVA, *F_(6,27.6)_* = 29.70, *P*<0.001; Tukey-Kramer HSD, α = 0.05). The D-scores, ranks and *^lp^HPI_β1_* for the development study are shown in Table 5.

### Two-Choice Study (C-Score): Flight Bioassay

**Figure 1:**
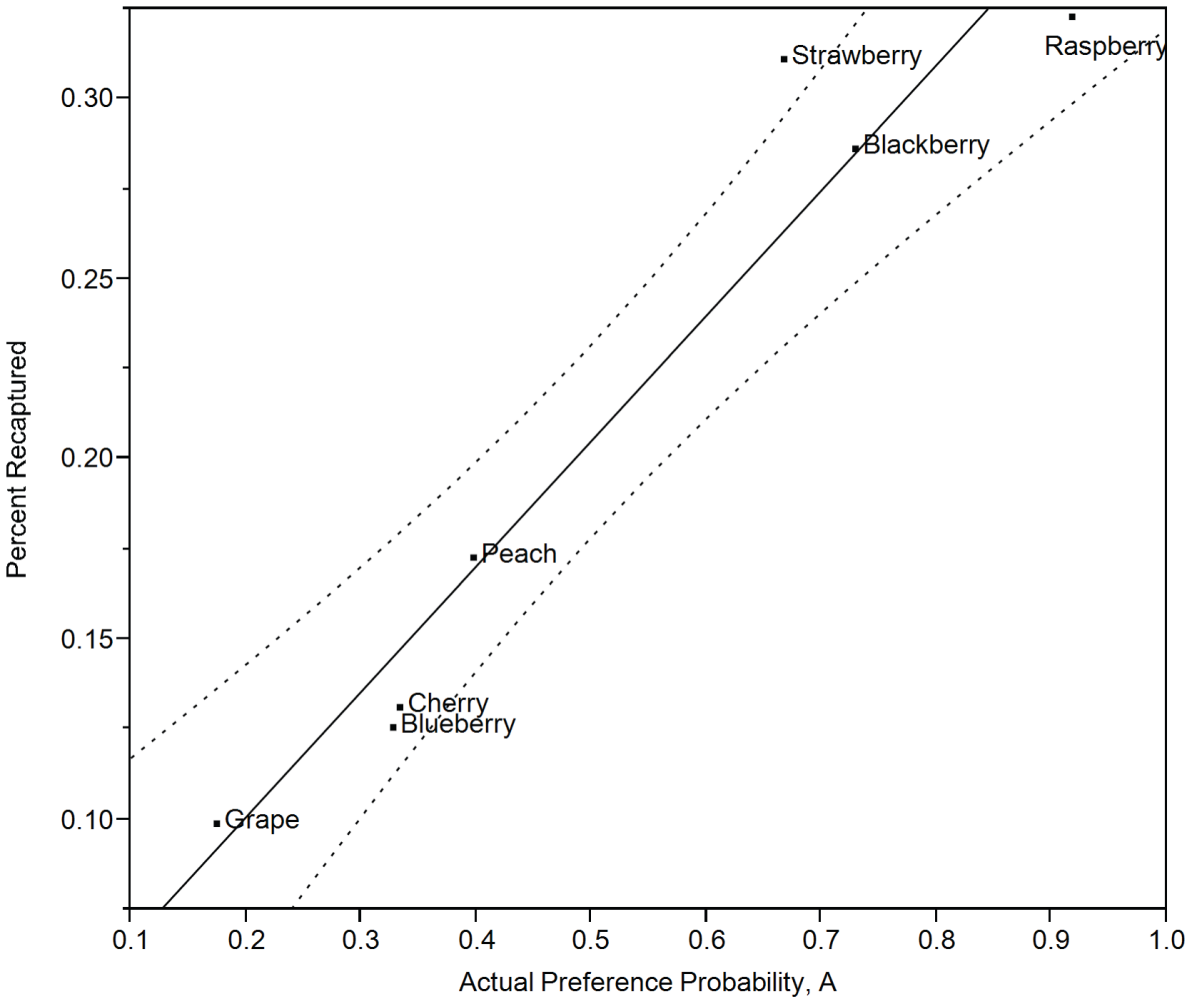
Correlation of spotted wing drosophila host volatile response to preference probabilities. Spotted wing drosophila attraction (i.e., recapture percentage) to host volatiles was evaluated in two-choice horizontal flight chamber bioasssays and correlated to the actual probability of preference, *A* (Eq. 1)(*R* ^2^ = 0.93, *F_(1,5)_* = 69.09, *P*<0.001). Dashed lines indicate 95% confidence interval for the correlation.

A total of 86 flight trials were conducted, in 21 pairwise combinations, with a minimum of 4 replicate trials for each combination. A total of 3440 mated female SWD were released and a total of 1410 were subsequently trapped (41% overall recapture). A strong correlation (*R* ^2^ = 0.93, *F_(1, 5)_* = 69.95, *P*<0.001) between recapture and corresponding actual preference probabilities, *A* (Eq. 1), (Fig. 1) supports actual probabilities as indicators of preferential attraction to host volatiles. Additionally, the use of actual preference probabilities instead of the mean number of recaptured SWD (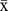 = 8.20, *s* = 7.15) minimized observed variation.

Mean female SWD recaptured in traps differed depending on host (raspberry, 12.92^a^; strawberry, 12.46^a^; blackberry, 11.46^a,b^; peach, 6.92^b,c^; cherry, 5.25^c^; blueberry, 5.04^c^; grape, 3.96^c^) (ANOVA: *F_(6,165)_* = 9.236, *P*<0.001). Mean SWD recaptured not connected by the same letter were significantly different (Tukey-Kramer HSD, α = 0.05). Raspberries have the highest actual preference probability (*A_rasp_* = 0.917), followed by blackberries (*A_bkb_* = 0.729), strawberries (*A_straw_* = 0.667), peaches (*A_peach_* = 0.396), cherries (*A_cher_* = 0.333), blueberries (*A_blb_* = 0.327), and grapes (*A_grap_* = 0.173). The C-scores, ranks, and *^flt^HPI_β_*_2_ for the Flight Bioassay are shown in Table 5.

#### Effect of pairing order

The 50-event simulation yielded C-scores for the 10 hosts that were subsequently indexed and ranked. Pairing order had a statistically significant effect on the resulting C-scores (*χ^2^*_128; 900_ = 2180.13; *p*<0.001). The range of variation, however, did not differ by sequence position (ANOVA; *F*_8,72_ = 0.5847; *p* = 0.7873), indicating that no specific position provided a scoring advantage over another. These results reinforce the use of a standard pairing order procedure for HPI analysis (highest WLT vs. lowest WLT method [*vide supra*]).

### Multiple-Choice Study 1 (M-Score): Population Level Oviposition

When presented concurrently with multiple hosts, populations of SWD females oviposited more frequently on strawberries than on other available hosts (Welch's ANOVA: *F_(6, 69.57)_* = 122.74, *P*<0.001; Tukey-Kramer HSD, α = 0.05) with mean adult SWD emergence (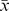
±*s*): strawberries, 26.45±10.78/berry; raspberries, 5.39±1.45/berry; cherries, 7.01±3.06/berry; blackberries, 6.56±3.74/berry; blueberries, 1.90±0.90/berry, grapes, 0.43±0.30/berry, and peaches, 0.04±0.20/fruit, suggesting females have the greatest preference for ovipositing in strawberries. M-scores corroborated strawberries were preferred over all fruit types, with strawberries accounting for 28.1% of all emerging adults when normalized for surface area as described above (Table 5). Raspberries, however, had a higher M-score than cherries and blackberries despite a lower mean emergence rate. This result reinforces the advantage of using M-scores over means to rank host preference, as means are sensitive to inclusion of replicates that are non-normal. The M-scores, ranks, and *^pop^HPI_β_*_3_ for the Population Oviposition study are shown in Table 5. The *^pop^HPI_β3_* ranking is generally consistent with the relative adult emergence of SWD from fruit hosts as described in the study of Lee et al. [27], which examined choice-based oviposition at the population level (<20 females).

### Multiple-Choice Study 2 (M-Score): Individual Level Oviposition

Adult emergence was not observed from any of the seven potential hosts in 40.7% of the replicated trials. When adult emergence was observed, data (Table 5) suggested strawberries were the most preferred ovipositional host averaging 5.11±0.78 offspring/female (ANOVA: *F_(6,187)_* = 6.746, *P*<0.001; Tukey-Kramer HSD, α = 0.05). While not statistically significant, it is interesting to note that no adult SWD emerged from peaches (Table 5). The M-scores, ranks, and *^ind^HPI_β4_* for the Individual Oviposition study are shown in Table 5.

### Host Potential Index and Ranking

Based on biological consideration(s) and/or precedence, researchers may adjust the weighting coefficients (*β*) in the HPI model to prioritize specific host preference or suitability studies.

All studies can be weighted equivalently. Applying this weighting arrangement in the context of SWD results, relative overall host potential values from Eq. 6 were: raspberries (*^tot^HPI_equal_* = 301.9; rank 1 of 7); strawberries (*^tot^HPI_equal_* = 298.1; rank 2 of 7); blackberries (*^tot^HPI_equal_* = 274.9; rank 3 of 7); cherries (*^tot^HPI_equal_* = 267.2; rank 4 of 7); peaches (*^tot^HPI_equal_* = 259.4; rank 5 of 7); blueberries (*^tot^HPI_equal_* = 251.7; rank 6 of 7); and grapes (*^tot^HPI_equal_* = 232.4; rank 7 of 7) (Table 5).

A single study may be emphasized. For example, both multiple-choice studies indicated a near zero potential for SWD to emerge from peaches, likely due to the inability of the ovipositor to penetrate the skin of commercially harvested fruit, at least fruit sourced from California for export distribution. If oviposition is emphasized (*β_lp_* = 0.05, *β_flt_* = 0.05, *β_pop_* = 0.45, *β_ind_* = 0.45), the *^tot^HPI_0.05,0.05,0.45,0.45_* would shift peaches to the host with the least potential (*^tot^HPI_0.05,0.05,0.45,0.45_* = 228.5; rank 7 of 7) while keeping the remaining hosts in order, respectively. On the other hand, if no-choice larval performance on fruit-agar diet was emphasized (e.g., *β_lp_* = 0.85, *β_flt_* = 0.05, *β_pop_* = 0.05, *β_ind_* = 0.05), peaches would reflect the highest relative potential to serve as SWD hosts (*^tot^HPI_0.85,0.05,0.05,0.05_* = 315.1; rank 1 of 7). The calculated HPI values could theoretically change by as much as 108 points for peaches depending upon prioritization (*min* = 221, *max* = 329) and lead to completely opposite conclusions regarding the potential of postharvest peaches to serve as hosts for SWD. Thus, the prioritization of weighting factors should be justified. In this example, no adults emerged from oviposition studies on intact peaches, so it would be questionable to use the development on fruit-agar material as the only HPI-criterion for evaluating the host potential of peaches relative to the other fruit.

**Figure 2:**
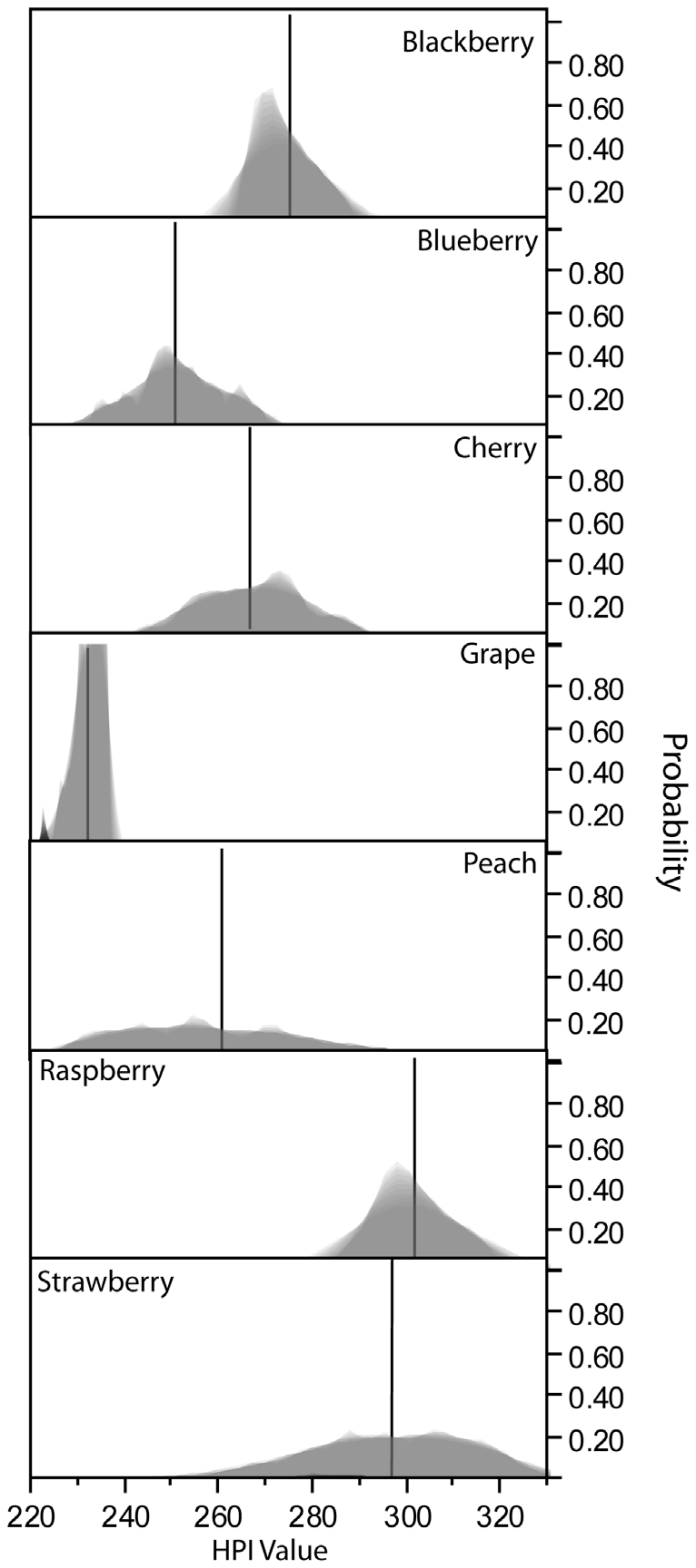
Potential HPI values when weighting coefficients are varied across four studies. The probability distributions of resultant Host Potential Index values derived from varying the weighting coefficieints (*β*) in the equation 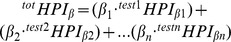
for the individual fruits shows the influence of coefficient weight selection. Vertical lines indicate the mean HPI value of each fruit.

Lastly, all weighting possibilities can be considered to account for variation resulting from prioritization. In the current study, *β*-coefficients (Eq. 6) were modulated (from 0.00 to 1.00) using the simplex-lattice design (*vide supra*); this allowed for differences in the relative host potential to be visualized and possible distinctions between hosts to be evaluated (Figure 2). For example, the distribution of HPI values for grapes were universally localized (*min* = 223, *max* = 236) near lower indexed values with relatively small deviation (*s* = 3.215). Values for raspberries, on the other hand, were localized at the higher end of the scale (*min* = 283, *max* = 326) and were also characterized by a small deviation (*s* = 8.393). As discussed above, HPI values for peaches show non-localized distribution (*min* = 221, *max* = 329) as characterized by a large deviation (*s* = 22.241). Mean total *^mean^HPI_varied_* (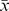
±*s*) values were calculated and compared for equivalency (Welch's ANOVA: *F_(6,678)_* = 3572.2, *P*<0.001): raspberry^a^ (301.8±8.39), strawberry^b^ (296.9±16.29), blackberry^c^ (274.8±6.64), cherry^d^ (267.4±12.15), peach^e^ (261.4±22.24), blueberry^f^ (251.4±10.96), and grape^g^ (232.2±3.21). *^mean^HPI_varied_* (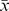
±*s*) values were then used as a quantifiable metric for distinguishing relative host potential of SWD in a postharvest scenario.

The HPI enables researchers to quantitatively probe the relationship between a host and the organism it harbors by combining results from disparate studies, having different designs, data ranges, values and units, or similar studies that potentially yield conflicting results [15], [47], [48]. Ultimately, the HPI can be used as a tool to enumerate how biotic/abiotic change (in the host or the organism being harbored) influences relative host potential by accommodating associated fluctuations in host acceptance thresholds [49] and fitness shifts that alter host selection [50]. Future research will evaluate how environmental and physiological states of the host and/or harbored organisms affect a single HPI or the comparison of multiple HPIs.

## Supporting Information

Supporting Information S1 **C-Score Calculations.** Step by step application for C-score analysis with example from two-choice study flight bioassay. Eqs. 1–3 are solved in detail.
(DOCX)

Supporting Information S2 **M-Score Calculations.** Step by step application for M-score analysis with example from multiple-choice population oviposition study. Eqs. 4–5 are solved in detail.
(DOCX)

Supporting Information S3 **HPI Table Derivation.** Rules and formulae for developing the Host Potential Index are presented and discussed.
(DOCX)
