# Supplementary material for: Quantifying Host Potentials: Indexing Postharvest Fresh Fruits for Spotted Wing Drosophila, Drosophila suzukii
Source: PLoS One. 2013 Apr 12;8(4):e61227. doi: 10.1371/journal.pone.0061227 (PMC3625224; doi:10.1371/journal.pone.0061227)
Supplement: Supporting Information S1 — C-Score Calculations. Step by step application for C-score analysis with example from two-choice study flight bioassay. Eqs. 1–3 are solved in detail. (DOCX) [file pone.0061227.s001.docx]

**S1. C-SCORE CALCULATIONS (EQUATIONS 1-3)**

Equations 1-3 were developed as an alternative method to evaluate two-choice studies involving three or more hosts. Eqs. 2-3 are derived from the Elo-rating formula used to rank chess players based on paired competition. Following is an example analysis using the results from the flight bioassay (two-choice study) focusing on raspberries as the potential host of interest.

**Step 1**: **Create a preference matrix**. Calculation of actual preference probability (or the win-loss-tie percentage) from multiple two-choice trials involving all variables begins with the creation of a preference matrix (Supplementary Table S1).

|  | **Flight Bioassay (olfactory response to hosts) W-L-T** | | | | | | | | | | | | | | | | | |  |  |  |
| --- | --- | --- | --- | --- | --- | --- | --- | --- | --- | --- | --- | --- | --- | --- | --- | --- | --- | --- | --- | --- | --- |
| Fruit | Blackberry | | | Blueberry | | | Cherry | | | Grape | | | Peach | | | Raspberry | | | Strawberry | | |
| Blackberry |  |  |  | 0 | 4 | 0 | 0 | 4 | 0 | 0 | 4 | 0 | 0 | 3 | 1 | 4 | 0 | 0 | 2 | 2 | 0 |
| Blueberry | 4 | 0 | 0 |  |  |  | 1 | 2 | 1 | 1 | 5 | 0 | 3 | 1 | 0 | 4 | 0 | 0 | 4 | 0 | 0 |
| Cherry | 4 | 0 | 0 | 2 | 1 | 1 |  |  |  | 1 | 2 | 1 | 4 | 0 | 0 | 3 | 1 | 0 | 1 | 3 | 0 |
| Grape | 4 | 0 | 0 | 5 | 1 | 0 | 2 | 1 | 1 |  |  |  | 2 | 2 | 0 | 4 | 0 | 0 | 4 | 0 | 0 |
| Peach | 3 | 0 | 1 | 1 | 3 | 0 | 0 | 4 | 0 | 2 | 2 | 0 |  |  |  | 4 | 0 | 0 | 4 | 0 | 0 |
| Raspberry | 0 | 4 | 0 | 0 | 4 | 0 | 1 | 3 | 0 | 0 | 4 | 0 | 0 | 4 | 0 |  |  |  | 1 | 3 | 0 |
| Strawberry | 2 | 2 | 0 | 0 | 4 | 0 | 3 | 1 | 0 | 0 | 4 | 0 | 0 | 4 | 0 | 3 | 1 | 0 |  |  |  |
| Totals | 17 | 6 | 1 | 8 | 17 | 1 | 7 | 15 | 2 | 4 | 21 | 1 | 9 | 14 | 1 | 22 | 2 | 0 | 16 | 8 | 0 |

**Supplementary Table S1. Flight Bioassay preference matrix**. The table presents the results from two-choice flight bioassays involving Host A (columns) versus a competing host, Host B (rows). The first sub-column for each host reports the number of times Host A was preferred (wins), the second sub-column reports the number of times Host A was not preferred (losses), and the third sub-column lists the number of times neither host was preferred (ties).

**Step 2: Apply Equation 1.** Calculate actual preference probability, *A_a_*, for each Host A – Host B pairing using Eq. 1. The number of times raspberries were preferred (wins), *P_a_*, is found in sub-column 1 of Supplementary Table S1, the number of times neither host was preferred (ties), *T_a_*, from sub-column 3, and the number of pairwise comparisons, *n* = 4 (*n* = 4 for all hosts, except for blueberries vs. grapes, *n* = 6). Each calculation for raspberries is shown in Table S2.

**Eq. 1**: *A_a_* = (*P_a_*+0.5*T_a_*)/*n*

| Competing potential host, Host B  (row) | *P_a_* = times raspberry preferred  (wins) | times raspberry not preferred (losses) | *T_a_* =  no preference  (ties) | *A_a_* = actual preference probability for raspberries vs opposing potential hosts  (winning percentage) |
| --- | --- | --- | --- | --- |
| Blackberry | 4 | 0 | 0 | (4 + 0.5(0))/ 4 = 4/4 = **1.000** |
| Blueberry | 4 | 0 | 0 | (4 + 0.5(0))/ 4 = 4/4 = **1.000** |
| Cherry | 3 | 1 | 0 | (3 + 0.5(0))/ 4 = 3/4 = **0.750** |
| Grape | 4 | 0 | 0 | (4 + 0.5(0))/ 4 = 4/4 = **1.000** |
| Peach | 4 | 0 | 0 | (4 + 0.5(0))/ 4 = 4/4 = **1.000** |
| Strawberry | 3 | 1 | 0 | (3 + 0.5(0))/ 4 = 3/4 = **0.750** |
| **Overall** | **22** | **2** | **0** | (22 + 0.5(0))/ 24 = 22/24 = **0.917** |

**Supplementary Table S2: Equation 1 calculations**. Calculations for raspberries using Equation 1 and Table 1.

The resulting actual preference probabilities, *A_a_*, between each host in our two-choice Flight Bioassay is given in Supplementary Table S3 (See Table 2 in paper).

| **Fruit** | **Blackberry** | **Blueberry** | **Cherry** | **Grape** | **Peach** | **Raspberry** | **Strawberry** |
| --- | --- | --- | --- | --- | --- | --- | --- |
| Blackberry |  | 0.000 | 0.000 | 0.000 | 0.125 | 1.000 | 0.500 |
| Blueberry | 1.000 |  | 0.375 | 0.167 | 0.750 | 1.000 | 1.000 |
| Cherry | 1.000 | 0.625 |  | 0.375 | 1.000 | 0.750 | 0.250 |
| Grape | 1.000 | 0.833 | 0.625 |  | 0.500 | 1.000 | 1.000 |
| Peach | 0.875 | 0.250 | 0.000 | 0.500 |  | 1.000 | 1.000 |
| Raspberry | 0.000 | 0.000 | 0.250 | 0.000 | 0.000 |  | 0.250 |
| Strawberry | 0.500 | 0.000 | 0.750 | 0.000 | 0.000 | 0.750 |  |
| **Overall *A_a_*** | **0.729** | **0.327** | **0.333** | **0.173** | **0.396** | **0.917** | **0.667** |

**Supplementary Table S3. Actual preference probabilities from Flight Bioassay study.** Calculations for all pairwise combinations of hosts were evaluated using Eq. 1 and Table 1.

**Step 3**: **Determine calculation order for Eq 2-3**. Arrange potential hosts from highest overall *A_a_* to lowest. Here, raspberries have the highest overall actual preference probability (*A_rasp_* = 0.917) so will be designated as Host 1, followed by blackberries (*A_black_* = 0.729) Host 2, strawberries (*A_straw_* = 0.667) Host 3, peaches (*A_peach_* = 0.396) Host 4, cherries (*A_cherry_* = 0.333) Host 5, blueberries (*A_blue_* = 0.327) Host 6, and grapes (*A_grape_* = 0.173) Host 7. This is the order in which we will apply Eq. 2-3.

**Step 4**: **Calculate expected preference**. Calculation of expected preference probability, *E_a_*, (or how many wins would we expect) from multiple two-choice trials involving all hosts, begins with the highest preference probability versus the lowest preference probability, or in our paper, raspberries (Host 1) vs. grapes (Host 7). All unevaluated hosts initially begin with a C-score of *C* = 900. So *C_a_* = *C_rasp_* = 900, and *C_b_* = *C_grape_* = 900.

**Eq. 2**.

Thus, *E_a_* for raspberries would be

As we would expect, two apparently equal opponents should have an equal chance of being preferred (or winning). But we know from our studies that raspberries are actually much more strongly preferred than grapes – 100% of the time, in fact.

**Step 5:** **Calculate C-Scores for Host 1**. We can use the knowledge of actual and expected preference to calculate a new C-Score for both raspberries and grapes using Eq. 3, the modified Elo-rating formula. Raspberries and grapes were paired against each other *n* = 4 times. From our C-Score/K-Factor table (Table 1 in main article), we know that C-Scores of 900 have a K-Factor of 36. The root of *n* times the K-Factor tells us how many potential points are available for C-Score modification. As hosts achieve higher or lower C-Scores, the points available for re-scoring (K-Factor) also change. Always processing Host 1 first, we know that *C_0_* = 900 for raspberries, *n* = 4 trials, K = 36, *A_a_* = 1.00, and *E_a_* = 0.500. We put these into Eq. 3 to determine the resulting C-Score for raspberries after being paired against grapes.

**Eq. 3**. $C_{1}=C_{0}+(\sqrt{n}\cdot\text{K})(A_{a}-E_{a})=900+(2*36)(1.00-0.500)=900+72(0.500)=900+36=936$

The resulting C-Score for raspberries is now 936. This value will be used in the subsequent pairing against Host 6, in our case, blueberries (*C_0_* = 900).

**Step 6**: **Calculate C-Scores for Host n**. Before proceeding, we need to calculate the C-Score for Host 7, grapes. We know that *C_0_* = 900, *n* = 4 trials, K = 36, *A_a_* = 0.000, and *E_a_* = 0.500.

$$C_{1}=C_{0}+(\sqrt{n}\cdot\text{K})(A_{a}-E_{a})=900+(2*36)(0.00-0.500)=900+72(-0.500)=900-36=864$$

Here, the new C-Score for grapes is 864. In essence, the grapes were penalized for being preferred less than expected, and the raspberries benefited by being preferred more than expected. Further grape calculations are not done at this time, while raspberry calculations proceed to the next host, blueberries, where **Steps 3-5** are repeated. Understand that because raspberries (*C* = 936) now have a higher C-Score than blueberries (*C* = 900), raspberries will have a higher expected preference probability (from Eq. 2; *E_a_* = 0.552) than they did initially against grapes. The rest of the calculations for raspberries are shown in Supplementary Table S4.

| **Before / after calc. for C-scores** | 1^st^ calculation - Grapes | | 2^nd^ calculation - Blueberries | | 3^rd^ calculation- Cherries | | 4^th^ calculation - Peaches | | 5^th^ calculation - Strawberries | | 6^th^ calculation - Blackberries | |
| --- | --- | --- | --- | --- | --- | --- | --- | --- | --- | --- | --- | --- |
|  | Before | After | Before | After | Before | After | Before | After | Before | After | Before | After |
| **Raspberries** | 900.00 | 936.00 | 936.00 | 968.28 | 968.28 | 979.29 | 979.29 | 1007.21 | 1007.21 | 1014.24 | 1014.24 | **1038.13** |
| **Host *n*** | 900.00 | 864.00 | 900.00 | 867.72 | 900.00 | 888.99 | 900.00 | 872.08 | 900.00 | 892.97 | 900.00 | 876.11 |

**Supplementary Table S4. C-Score Calculations for Raspberry**. Before and after C-Scores for each raspberry – Host *n* pairing using Eq. 2-3.

The entire set of results for Eqs. 2-3 calculations for all hosts, is shown in Supplementary Table S5.

| Order of Calc.  Host Pairings | 1^st^ | 2^nd^ | 3^rd^ | 4^th^ | 5^th^ | 6^th^ | **Final**  **C-score** |
| --- | --- | --- | --- | --- | --- | --- | --- |
|  | Grape (0.173) | Blueberry (0.327) | Cherry (0.333) | Peach (0.396) | Strawberry (0.667) | Blackberry (0.729) |  |
| **Raspberry (0.917) (A)** | 936.00 | 968.28 | 979.29 | 1007.21 | 1014.24 | 1038.13 | **1038.13** |
| Host (B) | 864.00 | 867.72 | 888.99 | 872.08 | 892.97 | 876.11 |  |
| *n* pairings | *4* | *4* | *4* | *4* | *4* | *4* |  |
| **Second step calculations (Most preferred host removed)** | | | | | | | |
| **Blackberry (0.729) (A)** | 910.86 | 942.41 | 972.92 | 989.76 | 980.25 |  | **980.25** |
| Host (B) | 829.25 | 836.17 | 858.24 | 855.24 | 902.48 |  |  |
| *n* pairings | *4* | *4* | *4* | *4* | *4* |  |  |
| **Third step calculations (Top two most preferred hosts removed)** | | | | | | | |
| **Strawberry (0.677) (A)** | 931.00 | 957.41 | 929.43 | 957.86 |  | | **957.86** |
| Host (B) | 800.73 | 809.76 | 886.46 | 826.81 |  |  |  |
| *n* pairings | *4* | *4* | *4* | *4* |  |  |  |
| **Fourth step calculations (Top three most preferred hosts removed)** | | | | | | | |
| **Peach (0.396) (A)** | 824.19 | 840.24 | 881.00 |  | | | **881.00** |
| Host (B) | 803.35 | 793.71 | 845.70 |  |  |  |  |
| *n* pairings | *4* | *4* | *4* |  |  |  |  |
| **Fifth step calculations (Top four most preferred hosts removed)** | | | | | | | |
| **Cherry (0.333) (A)** | 850.33 | 835.51 |  | | | | **835.51** |
| Host (B) | 798.72 | 808.53 |  |  |  |  |  |
| *n* pairings | *4* | *4* |  |  |  |  |  |
| **Final step calculations (Remaining two hosts)** | | | | | | | |
| **Blueberry (0.327) (A)** | 835.87 |  | | | | | **835.87** |
| **Grape (0.173)** (B) | 771.38 |  |  |  |  |  | **771.38** |
| *n* pairings | *6* |  |  |  |  |  |  |

**Supplementary Table S5. Entire set of C-Score calculations for Flight Bioassay**. The resulting C-Scores for each pairwise comparison amongst seven potential spotted wing drosophila hosts are presented. Results shown were calculated from left to right, top to bottom.
