# Supplementary material for: Quantifying Host Potentials: Indexing Postharvest Fresh Fruits for Spotted Wing Drosophila, Drosophila suzukii
Source: PLoS One. 2013 Apr 12;8(4):e61227. doi: 10.1371/journal.pone.0061227 (PMC3625224; doi:10.1371/journal.pone.0061227)
Supplement: Supporting Information S2 — M-Score Calculations. Step by step application for M-score analysis with example from multiple-choice population oviposition study. Eqs. 4–5 are solved in detail. (DOCX) [file pone.0061227.s002.docx]

**S2. M-SCORE CALCULATIONS (EQUATIONS 4-5)**

In multiple-choice studies where the behavior of an organism towards a suite of potential hosts is evaluated it is important to ensure analyses reflect the preference of the organism. Many studies use statistical procedures that analyze the mean results of a behavioral response (e.g., mean eggs oviposited, mean number of hosts parasitized, etc.) which tend, by nature of the procedures, to devalue the order of preference of the organism. An example of this is provided in the main article. The M-Score attempts to reflect a level of preference in the analysis. Below is a detailed description of how to calculate M-Scores.

**Step 1: Determine “votes of preference.”** To begin analysis, data should be organized to reflect host preference. In our population oviposition study, for example, we were able to simultaneously expose all seven hosts to large populations of spotted wing drosophila (25 samples for each host, 4 replicates). Looking at the resulting adult emergence from equivalent samples, we created a table of mean SWD emerging from each sample (Supplementary Table S6).

| Sample # | Blackberry | Blueberry | Cherry | Grape | Peach | Raspberry | Strawberry |
| --- | --- | --- | --- | --- | --- | --- | --- |
| 1 | 6.3 | 4.8 | 16.1 | 2.7 | 0.0 | 16.8 | 30.6 |
| 2 | 19.4 | 8.0 | 15.7 | 3.4 | 0.0 | 16.6 | 23.2 |
| 3 | 13.8 | 4.5 | 17.0 | 3.6 | 0.0 | 21.4 | 12.7 |
| 4 | 21.2 | 5.5 | 17.7 | 3.8 | 0.0 | 20.1 | 19.5 |
| 5 | 31.5 | 8.8 | 17.9 | 3.6 | 0.0 | 18.9 | 25.3 |
| 6 | 12.2 | 8.5 | 15.6 | 2.4 | 0.0 | 20.2 | 31.1 |
| 7 | 15.8 | 5.8 | 11.3 | 1.8 | 0.0 | 20.5 | 16.6 |
| 8 | 17.1 | 5.0 | 19.9 | 1.4 | 0.0 | 19.3 | 18.3 |
| 9 | 10.8 | 8.8 | 22.4 | 1.6 | 0.0 | 11.1 | 27.5 |
| 10 | 16.2 | 7.8 | 10.8 | 2.2 | 0.0 | 17.1 | 17.9 |
| 11 | 22.0 | 5.3 | 15.8 | 0.9 | 0.0 | 21.8 | 25.6 |
| 12 | 14.7 | 9.0 | 13.4 | 1.0 | 0.0 | 14.8 | 25.0 |
| 13 | 9.6 | 7.8 | 16.3 | 0.8 | 0.0 | 14.5 | 19.7 |
| 14 | 19.5 | 9.3 | 35.3 | 2.2 | 1.0 | 17.7 | 23.9 |
| 15 | 8.9 | 10.3 | 12.8 | 1.5 | 0.0 | 25.9 | 14.5 |
| 16 | 14.1 | 7.3 | 11.3 | 3.3 | 0.0 | 18.8 | 9.4 |
| 17 | 17.2 | 3.5 | 9.4 | 1.3 | 0.0 | 11.4 | 29.8 |
| 18 | 24.3 | 3.5 | 7.0 | 1.5 | 0.0 | 15.4 | 10.5 |
| 19 | 6.2 | 3.5 | 9.5 | 2.3 | 0.0 | 10.1 | 23.8 |
| 20 | 9.4 | 4.5 | 12.8 | 0.8 | 0.0 | 12.9 | 18.7 |
| 21 | 3.8 | 1.8 | 9.2 | 0.8 | 0.0 | 11.5 | 14.1 |
| 22 | 5.7 | 4.8 | 10.9 | ***0.0*** | ***0.0*** | 11.0 | 35.6 |
| 23 | 2.5 | 3.3 | 9.6 | ***0.0*** | ***0.0*** | 14.0 | 11.0 |
| 24 | 1.6 | 1.5 | 7.0 | ***0.0*** | ***0.0*** | 14.8 | 5.2 |
| 25 | 4.4 | 0.3 | 5.9 | ***0.0*** | ***0.0*** | 8.1 | 6.7 |
| Total | 163.9 | 47.5 | 175.2 | 10.7 | 1.0 | 134.7 | 661.4 |
| Std dev | 3.74 | 0.90 | 3.06 | 0.30 | 0.20 | 1.45 | 10.78 |
| Mean | 6.56 | 1.90 | 7.01 | 0.43 | 0.04 | 5.39 | 26.45 |

**Supplementary Table S6. Mean adult emergence per sample**. The mean number of spotted wing drosophila emerging from samples simultaneously exposed to large populations (and standardized by surface area) are reported along with totals, standard deviations, and means for all hosts.

Using common analytical methods, in this case an ANOVA, we would come to the conclusion that strawberries were the most preferred, followed by the blackberry-cherry-raspberry group, blueberries, and concluding with the grape-peach grouping (Table 5 in main article). The mean values reported here, however, do not reflect host preferences from each sample.

To gather preference information, hosts are ranked from high (1) to low (7) for each sample. This determines the preference order for each sample (Supplementary Table S7). For example, the “votes of preference” by the ovipositing females for sample #3 were raspberries, cherries, blackberries, strawberries, blueberries, grapes, and peaches coming in last.

| Sample # | Blackberry | Blueberry | Cherry | Grape | Peach | Raspberry | Strawberry |
| --- | --- | --- | --- | --- | --- | --- | --- |
| 1 | 4 | 5 | 3 | 6 | 7 | 2 | 1 |
| 2 | 2 | 5 | 4 | 6 | 7 | 3 | 1 |
| 3 | 3 | 5 | 2 | 6 | 7 | 1 | 4 |
| 4 | 1 | 5 | 4 | 6 | 7 | 2 | 3 |
| 5 | 1 | 5 | 4 | 6 | 7 | 3 | 2 |
| 6 | 4 | 5 | 3 | 6 | 7 | 2 | 1 |
| 7 | 3 | 5 | 4 | 6 | 7 | 1 | 2 |
| 8 | 4 | 5 | 1 | 6 | 7 | 2 | 3 |
| 9 | 4 | 5 | 2 | 6 | 7 | 3 | 1 |
| 10 | 3 | 5 | 4 | 6 | 7 | 2 | 1 |
| 11 | 2 | 5 | 4 | 6 | 7 | 3 | 1 |
| 12 | 3 | 5 | 4 | 6 | 7 | 2 | 1 |
| 13 | 4 | 5 | 2 | 6 | 7 | 3 | 1 |
| 14 | 3 | 5 | 1 | 6 | 7 | 4 | 2 |
| 15 | 5 | 4 | 3 | 6 | 7 | 1 | 2 |
| 16 | 2 | 5 | 3 | 6 | 7 | 1 | 4 |
| 17 | 2 | 5 | 4 | 6 | 7 | 3 | 1 |
| 18 | 1 | 5 | 4 | 6 | 7 | 2 | 3 |
| 19 | 4 | 5 | 3 | 6 | 7 | 2 | 1 |
| 20 | 4 | 5 | 3 | 6 | 7 | 2 | 1 |
| 21 | 4 | 5 | 3 | 6 | 7 | 2 | 1 |
| 22 | 4 | 5 | 3 | ***6*** | ***6*** | 2 | 1 |
| 23 | 5 | 4 | 3 | ***6*** | ***6*** | 1 | 2 |
| 24 | 4 | 5 | 2 | ***6*** | ***6*** | 1 | 3 |
| 25 | 4 | 5 | 3 | ***6*** | ***6*** | 1 | 2 |

**Supplementary Table S7. “Votes of preference” based on mean adult emergence across each sample**. Note the values in bold red italics in both tables above. In cases of ties, we cannot determine which was preferred, so equivalent hosts are given the next ranking. Here, grape and peach samples 22-25 each had no adult emergence and were assigned a “voting preference” of 6^th^.

**Step 2. Determine weighting factors for each place**. The weighting factor, Eq. 4, is independent of the data. In our study, we have 7 hosts, *h* = 7, so our weighting factor for first place (*j* = 1) is as follows:

**Eq. 4**. $W_{j}=\frac{h^{-1}}{j(j-1)!}$, therefore $W_{j}=\frac{h^{-1}}{j(j-1)!}=\frac{7^{-1}}{1(1-1)!}=\frac{7^{-1}}{1(0)!}=\frac{7^{-1}}{1}=\frac{0.1429}{1}=0.142857$

For second place, *j* = 2,

$$W_{j}=\frac{h^{-1}}{j(j-1)!}=\frac{7^{-1}}{2(2-1)!}=\frac{7^{-1}}{2(1)!}=\frac{7^{-1}}{2}=\frac{0.1429}{2}=0.071429$$

For subsequent places, *W_3_* = 0.02381, *W_4_* = 0.005952, *W_5_* = 0.00119, *W_6_* = 0.000198, and *W_7_* = 0.0000283.

**Step 3. Tabulate 1^st^ place votes, 2^nd^ place votes, etc. for each host.**  Using the data from the Table S7, a new table summarizing the number of 1^st^ place votes, 2^nd^ place votes, etc., for each host, is created (Supplementary Table S8).

|  | **1st** | **2nd** | **3rd** | **4th** | **5th** | **6th** | **7th** | **Total Votes (*N*)** |
| --- | --- | --- | --- | --- | --- | --- | --- | --- |
| **Blackberry** | 3 | 4 | 5 | 11 | 2 | 0 | 0 | 25 |
| **Blueberry** | 0 | 0 | 0 | 2 | 23 | 0 | 0 | 25 |
| **Cherry** | 2 | 4 | 10 | 9 | 0 | 0 | 0 | 25 |
| **Grape** | 0 | 0 | 0 | 0 | 0 | 25 | 0 | 25 |
| **Peach** | 0 | 0 | 0 | 0 | 0 | 4 | 21 | 25 |
| **Raspberry** | 7 | 11 | 6 | 1 | 0 | 0 | 0 | 25 |
| **Strawberry** | 13 | 6 | 4 | 2 | 0 | 0 | 0 | 25 |
| **Total** | 25 | 25 | 25 | 25 | 25 | 29 | 21 |  |

**Supplementary Table S8. Tabulated “votes of preference” (*V*) for each host.**

**Step 4: Calculate M-Scores.** At this point we are ready to calculate M-Scores for each host. Using blackberries as the first example, we know the weighting factor, *W_j_*, for each place (Step 2), the number of votes for each place and host, *V_ij_* (Step 3) (Supplementary Table S8), and the number of overall votes for each host, *N_i_* (Step 3)(Supplementary Table S8). Using **Eq. 5**, we calculate the score for each place (1^st^ – 7^th^) for blackberries and sum them together for a total M-Score.

Blackberry 1^st^ place

**Eq. 5.** $M_{i}=\sum_{j=1}^{h} \left(\frac{W_{j}V_{ij}^{2}}{N_{i}}\right)$, therefore $M_{i}=\sum_{j=1}^{h} \left(\frac{W_{j}V_{ij}^{2}}{N_{i}}\right)=\left(\frac{0.142857*3^{2}}{25}\right)=\left(\frac{1.285713}{25}\right)=0.05142852$

Blackberry 2^nd^ place

$$M_{i}=\sum_{j=1}^{h} \left(\frac{W_{j}V_{ij}^{2}}{N_{i}}\right)=\left(\frac{0.071429*4^{2}}{25}\right)=\left(\frac{1.142864}{25}\right)=0.045714$$

3^rd^ place = 0.02381, 4^th^ place = 0.02881, 5^th^ place = 0.00019, 6^th^ place = 0, and 7^th^ place = 0. Summing these together, *M_blackberry_* = 0.051429 + 0.045714 + 0.02381 + 0.02881 + 0.00019 + 0 + 0 = 0.149952. The process is then repeated for blueberries, cherries, etc. until all hosts have been scored. The calculated place results are listed in detail (Supplementary Table S9) and summarized in Table 5 of the main article.

|  | 1^st^ | 2^nd^ | 3^rd^ | 4^th^ | 5^th^ | 6^th^ | 7^th^ | *M-Score (total)* | Rank |
| --- | --- | --- | --- | --- | --- | --- | --- | --- | --- |
| Blackberry | 0.051429 | 0.045714 | 0.02381 | 0.02881 | 0.00019 | 0 | 0 | **0.150** | 4 |
| Blueberry | 0 | 0 | 0 | 0.000952 | 0.02519 | 0 | 0 | **0.026** | 5 |
| Cherry | 0.022857 | 0.045714 | 0.095238 | 0.019286 | 0 | 0 | 0 | **0.183** | 3 |
| Grape | 0 | 0 | 0 | 0 | 0 | 0.00496 | 0 | **0.005** | 6 |
| Peach | 0 | 0 | 0 | 0 | 0 | 0.000127 | 0.0005 | **0.001** | 7 |
| Raspberry | 0.28 | 0.345714 | 0.034286 | 0.000238 | 0 | 0 | 0 | **0.660** | 2 |
| Strawberry | 0.965714 | 0.102857 | 0.015238 | 0.000952 | 0 | 0 | 0 | **1.085** | 1 |

**Supplementary Table S9. Calculated place values from M-Scores (Eq. 5) for each host and place**. The total M-Score (the sum of all places) and subsequent rank are also provided.
