# Supplementary material for: Quantifying Host Potentials: Indexing Postharvest Fresh Fruits for Spotted Wing Drosophila, Drosophila suzukii
Source: PLoS One. 2013 Apr 12;8(4):e61227. doi: 10.1371/journal.pone.0061227 (PMC3625224; doi:10.1371/journal.pone.0061227)
Supplement: Supporting Information S3 — HPI Table Derivation. Rules and formulae for developing the Host Potential Index are presented and discussed. (DOCX) [file pone.0061227.s003.docx]

**S3. HPI TABLE DERIVATION**

The Host Potential Index is tabulated so that columnar values correspond to the number of hosts compared in the study and row values represent host ranks. The table provides indexed values for comparative studies involving 2 to 20 hosts. If more than 20 hosts were evaluated simultaneously, a more comprehensive table would need to be developed. In a literature search, however, no single comparative study exceeding 20 hosts could be found.

Indexed values were derived through an iterative process that conformed to three rules: 1) no value can reflect a “zero” potential; 2) the spread of values should be positively correlated to both rankings and the number of hosts evaluated (e.g., 2^nd^ of 14 hosts results in a stronger host potential than being 2^nd^ of 5 hosts); and, 3) the table should have a 20 host maximum. Using these three rules as guidelines, a formula was generated using the number of hosts in a study, *h*, and the rank of a particular host within that study, *R*, that produced indexed values were used to seed the HPI table when rounded to the nearest whole number.
